# The Practice Environment—How Coaches May Promote Athlete Learning

**DOI:** 10.3389/fspor.2022.957086

**Published:** 2022-07-22

**Authors:** Paul Larkin, James Barkell, Donna O'Connor

**Affiliations:** ^1^Institute for Health and Sport, Victoria University, Melbourne, VIC, Australia; ^2^Maribrynong Sports Academy, Melbourne, VIC, Australia; ^3^Sydney School of Education and Social Work, The University of Sydney, Sydney, NSW, Australia

**Keywords:** talent development, youth, coaching, training, microstructure

## Abstract

The coaching environment is the primary teaching and learning medium for the development of athlete skills. Therefore, by understanding how practice environments are designed to facilitate learning, coaches can make decisions around the structure of specific activities and behavior to promote athlete learning and development. This short review examines the coaching environment literature, with a particular focus on the structure and content within a practice session. The review will highlight the specific activities coaches utilize to develop athletes technical and tactical skills. Further, the coaching behaviors used to promote athlete learning is discussed, and how coach athlete interactions may influence learning. Finally, we provide applied recommendations for coaches, and highlight areas for future coaching science research.

## Introduction

The development of sporting expertise is associated with the engagement in a range of sport-specific activities that aid athlete development. To investigate this, researchers have used cross-sectional retrospective recall techniques, to identify the types of activities and the associated time invested in them by high performing athletes compared to their lower level counterparts (i.e., intermediate; novice). Findings have revealed a variety of sport-specific activities, which contribute to athlete performance, including primary sport coach-led practice; primary sport peer-led play and other sport practice and play (Güllich, 2019; Güllich et al., 2021). However, during this period of development, researchers have indicated one of the central factors in athlete growth is coach-led practice as time invested in this type of practice differentiates high-performing and lower skilled individuals (Güllich, 2019; Barth et al., 2020). While these findings highlight the importance of investing time in certain activities, such as coach-led practice, there is still limited knowledge regarding the micro-structure of these sessions and how they may contribute to athlete development. It should be noted, while the findings do not discount the importance of other activities, such as peer-led play, the current review paper aims to provide an overview of coach-led practice. Specifically, the elements within a session including the structure and behaviors used by coaches is examined, followed by practical implications and future research directions. It should be noted, the literature reviewed in the following section provides an overview of the key findings. Within the studies the participants, both athletes and coaches, may have been either male or female. As the papers reviewed do not provide gender based differences, we do not believe it is imperative to differentiate between male and female participants. Thus, the findings and recommendations can be applied for both genders.

## Micro-Structure of Practice

A key element of the motor learning literature is understanding the importance of practice structure on the acquisition of motor skills during practice (e.g., Barreiros et al., 2007; Spittle, 2013; Broadbent et al., 2015). This is especially true as the coaching environment is the primary teaching and learning medium for the development of players' technical and tactical skills (Cushion and Jones, 2001; Ford et al., 2010; Partington and Cushion, 2013). By determining how practice environments should be designed to facilitate learning, coaches and practitioners will be more aware of how activities should be designed to facilitate skill development (Roca and Ford, 2020). However, there is limited understanding of the specific practice structures and pedagogies coaches use across a range of sports and contexts (Kinnerk et al., 2019). Determining the underlying structure of practice sessions will inform the coaching process and provide insight into current coaching philosophy and pedagogical approaches (Hüttermann et al., 2014; Kinnerk et al., 2019). One method used to determine the structure of coaching sessions, is *via* systematic observational tools which monitor the time invested in specific activities (Cushion and Jones, 2001; Ford et al., 2010; Partington and Cushion, 2013). Generally, researchers have aimed to describe the time invested in training form (i.e., activities focused on developing skills *via* drills and isolated activities performed in non-pressurized environments (Ford et al., 2010; Partington and Cushion, 2013; Partington et al., 2014); and playing form activities (i.e., activities that replicate the demands of the game *via* small-sided or conditioning games (Partington and Cushion, 2013).

Researchers exploring the microstructure of practice examined the breakdown of time invested in training and playing form activities. As shown in Table 1, researchers found a greater proportion of time was invested in training form activities (53–65% of practice time) compared to playing form activities (Ford et al., 2010; Harvey et al., 2013; Low et al., 2013; Partington and Cushion, 2013; Partington et al., 2014; Hall et al., 2016). This type of practice places an emphasis on isolated skill drills in non-pressured environments. However, more recent investigations have shown a shift, with studies in rugby and soccer indicating coaches developed sessions with more playing form activities (Hall et al., 2016; O'Connor et al., 2018a). While this is encouraging, O'Connor et al. (2018a), extended the previous literature by also analyzing periods of inactivity within a session (i.e., periods during a session where the team are not actively participating in either training or playing form activities) and found ~30% of session players were inactive as they listened to the coach.

**Table 1:** Comparison of the mean percentage of time invested in training form, playing form and inactivity across multiple examinations of youth coaching sessions.

			**Training form**		**Playing form**		**Inactivity**	
**References**	**Sport**	**Level of Competition**	**Mean**	**SD**	**Mean**	**SD**	**Mean**	**SD**
Ford et al. (2010)	Soccer	Youth - Elite	60.00	20.00	40.00	20.00		
		Youth - Sub-elite	65.00	22.00	35.00	22.00		
		Youth - Non-elite	72.00	15.00	28.00	15.00		
Low et al. (2013)	Cricket	Elite adolescents	85.00	11.00	0.00	0.00		
		Elite children	65.00	34.00	21.00	39.00		
		Recreational ADOLESCENTS	83.00	31.00	11.00	33.00		
		Recreational Children	41.00	37.00	45.00	43.00		
Partington and Cushion (2013)	Soccer	Youth - Elite	53.00		47.00			
Harvey et al. (2013)	Hockey	Collegiate	41.45	18.11	35.09	16.12		
	Volleyball	Collegiate	45.29	12.69	39.14	12.02		
	Basketball	Collegiate	40.5	13.66	35.74	15.35		
Partington et al. (2014)	Soccer	Under 10s & 11s	54		46			
		Under 12s & 13s	73		27			
		Under 14s & 15s	38		62			
O'Connor and Larkin (2017)	Mixed	Junior	45.69	23.16	26.39	19.30	26.55	12.35
		Youth	18.85	14.01	50.26	17.06	28.61	6.54
		Senior	28.89	12.22	52.04	12.49	19.07	3.00
Ford and Whelan (2016)	Soccer	Child	20.00	13.00	63.00	12.00	17.00	5.00
		Adolescent	21.00	14.00	56.00	14.00	23.00	7.00
Hall et al. (2016)	Rugby Union	Senior - Elite	41.50		58.50			
O'Connor et al. (2018a)	Soccer	Youth	22.30	13.40	40.90	14.80	36.80	9.80
Roca and Ford (2020)	Soccer	Youth	20.00	8.00	62.00	9.00	17.00	3.00
Fuhre and Sæther (2020)	Soccer	Youth Professional	44.3		55.7			
		Youth Non-Professional	36.7		63.3			
Ahmad et al. (2021)	Soccer	Elite - Youth	46.8		34.7		18.5	
		Non-Elite Youth	45		36.6		18.4	

In relation to the specific sequencing of the session, researchers have found sessions are structured to provide training form activities (i.e., individual and paired activities; drills) at the start of the session and then progressed to more playing form activities (i.e., small-sided games then larger games) later in the session (O'Connor et al., 2018a; Kinnerk et al., 2019). For example, early in a session, coaches prescribe more individual or drill based activities (i.e., training form), where there is an emphasis on either skill execution or conditioning. As the session progresses, there is a decrease in the use of drills and individual activities, counteracted by increased use of modified, small and larger sided games (i.e., playing form) (O'Connor et al., 2018a). Interestingly, the micro-structure of practice may differ depending on competition level or athlete ability. O'Connor and Larkin (2017) investigated the activities conducted in practice sessions across a range of sports (i.e., soccer, rugby union, rugby league and Australian Rules football) and age groups (senior—elite adult; youth - Under 16/18; junior - Under 10/12). Results found significantly more periods of training form and less time allocated to playing form activities for junior athletes compared to youth and senior athletes. The findings demonstrate there is still an emphasis on drill-based activities at a junior level, with coaches less prone to incorporating game-based practice (26% of the session time). This difference in practice micro-structure was also demonstrated in professional and non-professional Norwegian U16 soccer teams (Fuhre and Sæther, 2020). The findings highlighted the non-professional teams used more playing form activities (63.3%) compared to the professional team (55.7%).

Studies have also examined the breakdown of activities conducted within a practice session, to provide a more detailed understanding of the use of playing and training form within practice sessions. O'Connor et al. (2017) found the greatest proportion of time within youth soccer practice sessions was allocated to large- (24.8%) and small-sided games (15.3%), with drill-based (15.1%), individual (5.4%), and paired activities (2.4%). Fuhre and Sæther (2020) examined the breakdown of the specific activities undertaken and found that training form was divided into fitness (i.e., improving individual fitness), technical (i.e., isolated technical drills) and skill (i.e., re-enactments of isolated game incidents, corner, free-kick) activities. While there were some similarities between the professional and non-professional club in the time allocated to fitness (18.3 and 13.4%) and technical (13 and 23.3%) activities, the non-professional club did not spend any time in skill activities, while the professional club spent 13% of time doing these activities. In relation to playing form, the sessions examined the time invested in small-sided games (i.e., match-play with reduced numbers), conditioned games (i.e., characteristics of small-sided games, but with variations in rules) and phase of play (i.e., unidirectional match play toward a single goal) activities. Findings revealed professional and non-professional clubs allocated similar proportions of time to small-sided games (14.3 and 26.9%) and conditioned games (28.7 and 36.4%), however, the non-professional club did not allocate any time to phase of play, while the professional club spent 12.7% of time completing this type of activity. Furthermore, when exploring the breakdown of activities within sessions, there were differences depending on the age group of the athletes (O'Connor and Larkin, 2017). Coaches of junior athletes prescribe sessions with more isolated skill activities (48.9%), followed by small-sided games (27.8%), drills (13.7%) and fitness (10.3%) activities. In comparison, youth coaches organize sessions with more tactical play (42.3%) and drills (30.3%), with less of a focus on isolated skill activities (13.7%), small-sided games (11.6%) and fitness (11.6%) activities. Whereas senior coaches structure sessions with more tactical play (41.6%), with the rest of the time divided between isolated skill activities (19.1%), fitness (16.5%), drills (13.8%), and small-sided games (8.8%). The data highlights the differences associated with how coaches at different levels of competition structure practice for athlete development (O'Connor and Larkin, 2017; O'Connor et al., 2018a; Fuhre and Sæther, 2020; Ahmad et al., 2021).

When considering the reason for the structure of a training session, Kinnerk et al. (2019) found Gaelic football coaches' use of playing and training form was dependent on the stage of the macro-structure of the athletes' program. Therefore, during pre-season more time was dedicated to training form activities, however, there was a shift in-season with more time within sessions dedicated to playing form activities. It was postulated this was due to coaches believing it was important to increase the players fitness levels during pre-season, and thus increased levels of conditioning activities during this period. However, in-season, where there are more fatigue related issues for game performance, less time was associated with individual conditioning activities. Instead, these would be incorporated within playing form activities (Kinnerk et al., 2019). The authors conclude that coaches value both training and playing form activities, and suggest the reason for high amounts of training form activities was to increase the number of skill repetitions completed, thus providing immediate performance improvements (Gabbett et al., 2009; Kinnerk et al., 2019).

## Coach Behavior During Practice Sessions

Another important component of the practice session to consider is the coach's behavior and its influence on athlete learning. Coaching behaviors, the communication and interactions between the athlete and coach, play an influential role in overall athlete performance, skill development and learning (Cushion, 2013; Partington and Walton, 2019). This is inclusive of instruction styles, modeling, feedback, questioning, and observation either during or outside of activity (Cushion and Jones, 2001; Cushion, 2010; Ford et al., 2010; Partington et al., 2014; Cope et al., 2017; O'Connor et al., 2018a). Coaching behaviors have been evaluated using the Coach Analysis Intervention System (CAIS) (Cushion et al., 2012b) or a modified version (Partington and Cushion, 2013; O'Connor et al., 2018b), and is designed to provide operational definitions of a variety of coaching behaviors and measure their incidence within a practice session. In this review we are focusing on instruction, feedback and questioning as these behaviors tend to be most observed and therefore reviewed thoroughly within the research (see Table 2) (Partington and Cushion, 2013; Partington et al., 2014; O'Connor et al., 2018a). While these coaching behaviors are classified as singular events, Cushion (2010) describes these behaviors as often overlapping and intertwined depending on the circumstances in which they are utilized.

**Table 2:** Descriptions and examples of the coaching behaviors of Instruction, feedback and questioning (adapted from CAIS, Cushion et al., 2012b; Partington et al., 2014).

**Behavioral category**	**Primary behavior**	**Definition**	**Example**
Instruction	Pre-instruction	Initial instructions and information provided prior to the activity starting.	“The aim of the next activity is…”
	Concurrent instruction	Training cues or directions to explicitly inform an athlete toward a certain action or behavior.	“move right”, “pass to (name)”, “take the shot”, “mark your player”
	Post-instruction	Information given after the execution of the desired action	“You should always take the shot when its available”
Feedback	Specific feedback—positive	Specific verbal statements that are positive or supportive that specifically provide information about the quality of performance.	“I liked the way you focused on the ball”
	Specific feedback—negative	Specific verbal statements that are negative or unsupportive that specifically provide information about the quality of performance.	“Come on, you need to stay focused on the ball”
	General feedback—positive	General verbal statements OR non-verbal gestures (either positive or supportive).	“good work” or “well done”
	General feedback—negative	General verbal statements OR non-verbal gestures (either negative or unsupportive).	“that was hopeless” or “that was horrendous”
	Corrective feedback	Corrective statements that contain information that specifically aim to improve the performance at the next attempt.	“try passing earlier next time”
Questioning	Divergent questions	Multiple responses/options—more open	“What did you notice about the space in the defensive zone?”
	Convergent questions	Limited number of correct answers/options—more closed	“Who was the player that was free in the attacking zone?” or “Was that pass the best option there?”

Research indicates the use of instruction dominates coaching behaviors within youth practice sessions (Cushion, 2010; Ford et al., 2010; Cushion et al., 2012a; Partington and Cushion, 2013; O'Connor et al., 2018a). However, the amount of instruction provided during sessions vary depending on a range of factors, including age and athlete ability (Ford et al., 2010; Partington et al., 2014). There is a moderate reduction in total instruction as athletes develop with age, with coaches explaining this shift being due to younger age athletes requiring more information to correct mistakes and ensure improvement compared to older athletes (Partington et al., 2014).

While instruction can be considered holistically, the instructions provided within a session can also be divided into three primary behaviors, pre-instruction; concurrent instruction; and post-instruction (Cushion et al., 2012b; Partington et al., 2014) providing a more transparent depiction of when instruction is being utilized in the practice session. Concurrent instruction tends to be the most used form of instruction accounting for significantly greater use than pre or post instruction (e.g., 20% concurrent v 11% pre v 3% post instruction for U14/15s; Partington and Cushion, 2013). Reasons for this might be that coaches tend to mimic other coaches and it becomes a learnt behavior (Partington and Walton, 2019). Coaches might also prefer to instruct in the present in the fear of forgetting to mention the point later (Partington and Cushion, 2013). A concern with becoming over reliant on concurrent instruction is that this behavior tends to be a more explicit method of instruction and may promote athlete dependency on the coach rather than athletes working it out for themselves. Athletes may benefit more from implicit and deeper levels of learning which could be promoted through thought-provoking behaviors such as questioning (Masters and Maxwell, 2004; Gebauer and Mackintosh, 2007). Coaches tend to use those behaviors that are associated with the perception of quality coaching (Jones et al., 2004; Partington et al., 2014; Cope et al., 2017). Anecdotally, there is the perception instruction also provides the coach with credibility in the sport, with more instruction being correlated with quality coaching. The desired result is more respect from the athletes (Potrac et al., 2002; Cushion, 2010).

Providing feedback is another common behavior coaches use (Cushion, 2010; O'Connor et al., 2018a; Partington and Walton, 2019). Positive feedback has been demonstrated to be related to task accomplishment within athlete groups and is considered a preferred coaching behavior (Cushion, 2010). Youth coaches have indicated a preference to using positive forms of feedback with negative feedback being the least used (Partington et al., 2014; O'Connor et al., 2018a). Although the dominant form of feedback tends to be general positive (Partington et al., 2014; O'Connor et al., 2018b), which promotes self-confidence, it provides little if any meaningful information pertaining to the athlete performance (Horn, 1987). Alternatively, corrective feedback which is deemed more task specific and relevant to athlete learning is used consistently less throughout training periods than general positive and even positive specific feedback (O'Connor et al., 2018a). Whilst keeping feedback positive is good for athlete motivation, corrective feedback improves learning and performance when provided alongside positive feedback (Tzetzis et al., 2008) and hence should be utilized more often in the athlete development environment.

Observations have identified a tendency for feedback delivery to be evenly distributed between concurrent (during activity) and post activity (Barkell and O'Connor, 2011). Furthermore, feedback is generally provided during periods of player inactivity such as the huddle or a “freeze” scenario (O'Connor et al., 2018a). Results identified that 16.5% of the total session was based on the “freeze” principle to provide feedback to the group in relation to where they had been positioned at a given moment (O'Connor et al., 2018a). The use of a huddle to listen to the coach accounted for 9.9% of the practice time. Whilst the huddle can provide clearer messaging due to a greater focus on the coach by the athletes, it is a questionable behavior to cease all activity if the feedback is only relevant for a fraction of the group. An alternative is to take the relevant athlete aside and provide specific feedback while the activity continues (O'Connor et al., 2022).

While the use of questioning as a key pedagogical practice is known (Partington and Cushion, 2013; O'Connor et al., 2022), studies have found coaches often do not apply this behavior effectively (Low et al., 2013). Several studies have examined the use of questioning, with reports of only 7–8% of total coach interactions coming in the form of questioning (Ford et al., 2010; Partington and Cushion, 2013; Partington et al., 2014). Furthermore, early studies identified greater use of convergent questioning (87%) compared with divergent questioning (13%) (Partington et al., 2014). However, recent studies (O'Connor et al., 2018a, 2022) reported a shift in coaching behavior with more questioning being utilized (i.e., an average 71 questions/session which equated to almost one per minute), with a balance of convergent (52.2%) and divergent (47.8%) questions being used (O'Connor et al., 2022). Of the divergent questions posed, only 7% asked athletes to problem solve. Questioning, especially divergent questioning, is believed to generate a more thoughtful and abstract understanding due to the deeper thought processes required to respond (Ford and Whelan, 2016; O'Connor et al., 2020) in comparison to instruction and general feedback. In fact using questioning as a form of feedback has been identified as being advantageous to learning (O'Connor et al., 2017, 2020).

## Discussion and Recommendations

As learning is non-linear (i.e., learning is not generally a continuous linear progression of behavior but rather involves sudden changes over time; (Kelso, 1995), creating practice environments for optimal athlete learning is challenging for coaches. This review highlights there is a shift to more playing form activities within a session, although the use of certain activities may also be influenced by when in the season the session occurs. The most frequently used coaching behavior was instruction suggesting a prescriptive and direct approach is taken by coaches, although there is evidence of a greater use of questioning in recent times. Therefore, based on the literature reviewed in this short review, several practical recommendations can be provided for coaches to apply in their daily practice. To create learning environments for their athletes, the coach must deliberately plan each practice session. This involves knowing your athletes' capabilities and their needs and deciding what to prioritize in the upcoming practice session (Muir et al., 2011). When coaches know their athletes, they can differentiate or individualize practice rather than following a “one size fits all” approach (Amorose, 2007). As coaches don't want athletes to become bored or complacent if the task is too easy, or panic if the task is beyond their capability, they should plan to push athletes beyond their comfort zone where they are “stretched”, for learning to take place. An example of differentiation in a mixed ability squad, is for coaches to vary the task constraints (e.g., different rules, participant numbers, and/or field dimensions will influence their movement patterns, and the time and space athletes have to make decisions and execute skills) that groups of athletes are participating in rather than all playing the same game (i.e., 4v4).

Coaches also need to be clear on what the aim is for their practice session. The aim of the practice session and intended learning outcomes will influence the structure of practice the coach devises [e.g., type of activities—training (drills, conditioning) or playing form (small or large-sided games, phases of play); technical, tactical, physical, biopsychosocial focus; variability of practice etc.] and the coaching behavioral strategies they decide to implement (e.g., amount of instructions; use of questions; when and how they provide feedback etc.) (Abraham et al., 2014; Kinnerk et al., 2021). For example, just prior to competition the coach may use more direct and explicit approaches during drills as the focus is on performance and confidence rather than learning (Otte et al., 2020). While a specific session aim is important, coaches also need to be flexible and adapt during the session to manage the complexity of athlete learning (Nash and Taylor, 2021).

In relation to the specific practice design, coaches will utilize a range of approaches to suit the session goal (Pill, 2021). One example is a constraints-led approach, where the coach is the “designer” and manipulates various constraints (i.e., player, task, and environment) to replicate key conditions of the performance environment (i.e., transitioning from defense to attack). This provides an opportunity for athletes to learn by adapting to the situation through guided discovery and solution finding (Davids et al., 2017; Woods et al., 2020a). The decision on what and how constraints will be manipulated will be influenced by the session goal, the specific affordances within the environment coaches want athletes to explore, and the skill capabilities of the athletes (Correia et al., 2019; Renshaw et al., 2020; Woods et al., 2020b). By creatively manipulating the constraints and setting representative problems, athletes are given the opportunity to interpret game-related cues, adapt to team-mates and the opposition, explore options, make decisions, and execute technical skills, all within one activity (Pinder et al., 2011; McKay and O'Connor, 2018). This less prescriptive approach by coaches allows athletes to explore the “how, why, where, and when”, experiment and make mistakes as they evaluate and identify appropriate decisions and actions to game situations (Correia et al., 2019; Renshaw and Chow, 2019). For example, by manipulating rules, number of participants, and pitch size, coaches can challenge athletes and scaffold learning while increasing the frequency of repetition, reducing the conscious control of movement, and promoting high levels of athlete engagement, ownership, autonomy and motivation (Hornig et al., 2016; Woods et al., 2020a).

This review suggests coaches are still prone to over coaching with players inactive and listening to the coach for substantial amounts of time. As coaches are constrained by the amount of time they have with their athletes, they need to consider strategies to reduce inactivity, so athletes have greater opportunities to engage in active practice. This could include reducing the amount of direct instruction (e.g., using analogies to direct athletes to an external focus of attention, Otte et al., 2020), using brief cues or prompts; allowing the activity to progress longer to see if athletes can correct their own errors or find solutions before stopping to ask questions and provide feedback (O'Connor et al., 2018b); and where appropriate, providing feedback on the run to individual athletes rather than stopping the activity. Coaches need to consider where they want to provide the feedback—either in a huddle which takes time but has the athletes' attention compared to athletes “freeze where you are” and whether all athletes can see and hear (O'Connor et al., 2018b). They are also encouraged to be mindful of the amount of feedback they give, with a “less is more” approach recommended (Otte et al., 2020; Mason et al., 2021). Coaches are encouraged to plan and scaffold questions to assist athlete learning, basing the type of question posed on their athletes' needs and the nature of the situation (i.e., what do they want to draw the athletes' attention to?), while providing enough time for athletes to respond or encouraging athletes to collaborate to devise solutions (Woods et al., 2020a; O'Connor et al., 2022). Coaches are also encouraged to reflect on-action (i.e., athlete learning, what worked well or didn't and why) to inform planning of the next practice session (Gilbert and Trudel, 2001).

## Conclusion and Future Directions

In summary, this review highlights the practice environment and the specific elements that can influence athlete learning. Overall, the micro-structure of practice and the activities used to promote learning need to be well-planned. There should be a clear goal for each activity. Coaches also need to consider how they communicate with their athletes to ensure they are interacting in a manner that enables athlete growth. To develop further understanding, researchers should focus attempts on evaluating the micro-structure of practice and coach behaviors regarding effectiveness in promoting the intended athlete learning outcomes. Few studies have examined the women's practice environment. Longitudinal intervention studies involving individual elements (e.g., use of questioning) may provide further understanding of athlete learning to inform coaching practice as holistic evaluations require challenging research designs (large sample size, matched participants, control group, etc.).

## Author Contributions

All authors listed have made a substantial, direct, and intellectual contribution to the work and approved it for publication.

## Conflict of Interest

The authors declare that the research was conducted in the absence of any commercial or financial relationships that could be construed as a potential conflict of interest.

## Publisher's Note

All claims expressed in this article are solely those of the authors and do not necessarily represent those of their affiliated organizations, or those of the publisher, the editors and the reviewers. Any product that may be evaluated in this article, or claim that may be made by its manufacturer, is not guaranteed or endorsed by the publisher.
